# Analysis of EpCAM positive cells isolated from sentinel lymph nodes of breast cancer patients identifies subpopulations of cells with distinct transcription profiles

**DOI:** 10.1186/bcr2922

**Published:** 2011-08-04

**Authors:** Siri Tveito, Kristin Andersen, Rolf Kåresen, Øystein Fodstad

**Affiliations:** 1 Department of Tumor Biology, Institute for Cancer Research, Norwegian Radium Hospital, Oslo University Hospital, PB 4953 Nydalen, 0424 Oslo, Norway; 2 Department of Breast and Endocrine Surgery, Institute for Clinical Medicine, Ullevaal Hospital, Oslo University Hospital, PB 4953 Nydalen, 0424 Oslo, Norway; 3 Institute of Clinical Medicine, Faculty of Medicine, University of Oslo, PB 1171 Blindern, 0318 Oslo, Norway

## Abstract

**Introduction:**

The presence of tumor cells in the axillary lymph nodes is the most important prognostic factor in early stage breast cancer. However, the optimal method for sentinel lymph node (SLN) examination is still sought and currently many different protocols are employed. To examine two approaches for tumor cell detection we performed, in sequence, immunomagnetic enrichment and RT-PCR analysis on SLN samples from early stage breast cancer patients. This allowed us to compare findings based on the expression of cell surface proteins with those based on detection of intracellular transcripts.

**Methods:**

Enrichment of EpCAM and Mucin 1 expressing cells from fresh SLN samples was achieved using magnetic beads coated with the appropriate antibodies. All resulting cell fractions were analyzed by RT-PCR using four chosen breast epithelial markers (hMAM, AGR2, SBEM, TFF1). Gene expression was further analyzed using RT-PCR arrays and markers for epithelial to mesenchymal transition (EMT).

**Results:**

Both EpCAM and Mucin 1 enriched for the epithelial-marker expressing cells. However, EpCAM-IMS identified epithelial cells in 71 SLNs, whereas only 35 samples were positive with RT-PCR targeting breast epithelial transcripts. Further analysis of EpCAM positive but RT-PCR negative cell fractions showed that they had increased expression of MMPs, repressors of E-cadherin, SPARC and vimentin, all transcripts associated with the process of epithelial to mesenchymal transition.

**Conclusions:**

The EpCAM IMS-assay detected tumor cells with epithelial and mesenchymal-like characteristics, thus proving to be a more robust marker than pure epithelial derived biomarkers. This finding has clinical implications, as most methods for SLN analysis today rely on the detection of epithelial transcripts or proteins.

## Introduction

The presence of metastatic deposits in the axillary lymph nodes is the most powerful predictor of survival in early stage breast cancer patients [1-3]. The sentinel lymph node (SLN) is defined as the first node or group of nodes receiving lymph from a tumor area, and the status of the SLN has been shown to reflect the presence of metastases in the axillary lymph nodes [4,5]. Reliable detection of micrometastatic cells in the SLN is, therefore, a subject of great clinical interest, and several different protocols aimed at identifying breast epithelial cells within the lymphatic basin are currently in use. Metastatic cells may exist in low concentrations, making their identification and isolation a difficult task. Studies have shown that extensive re-examination of presumably negative nodes will identify more positive specimens, but techniques using tissue sections are labor intensive if more detailed analysis is warranted [6-9]. However, several recent papers have concluded that even small cell deposits may be of clinical relevance, suggesting that a comprehensive examination would be worthwhile [10-13].

Our laboratory has for many years worked with immunomagnetic selection (IMS) using paramagnetic beads coated with antibodies against cell surface proteins for positive selection of tumor cells from cell suspensions [14-16]. The method is fast, sensitive and allows further molecular characterization of isolated live cells [17]. The choice of antibodies is decisive for the impact of the IMS method, as only cells expressing the targeted cell surface proteins will be captured by the magnetic beads.

For identification of epithelial-derived cells the epithelial cell adhesion molecule EpCAM is a commonly used target [18]. EpCAM is a transmembrane glycoprotein expressed by both normal and malignant cells of epithelial origin (for reviews see [19,20]), but over-expressed in many carcinomas. In a recent study, EpCAM was shown to be over-expressed on all breast cancer metastases relative to the matched primary tumor [21]. Mucin 1 (MUC1), a membrane bound glycosylated phosphoprotein predominantly expressed by epithelial cells, is suggested to be a marker for detection of breast-cancer cells not expressing EpCAM [22]. Mucin 1 is over-expressed in several human malignancies, especially adenocarcinomas (for a review see [23]).

Metastatic tumor cells may also be identified by RT-PCR which relies on the detection of intracellular gene transcripts. By carefully selecting genes expressed by the target tumor cells, but absent from the normal stroma, this method may allow very sensitive detection of small metastatic deposits.

The aim of our study was to analyze fresh SLN samples from early stage breast cancer patients using in sequence, IMS and RT-PCR techniques for identification of tumor cells [24]. IMS with anti-EpCAM and anti-Mucin 1 antibodies were used in parallel on disaggregated tissue from SLN to enrich for cells expressing these epithelial proteins. The IMS isolated cell fractions were then analyzed by RT-PCR targeting four epithelial cell associated transcripts; hMAM, AGR2, SBEM and TFF1. This allowed us to compare findings based on the expression of external cell-surface proteins (IMS) with those based on intracellular transcripts (RT-PCR), and to elucidate the molecular heterogeneity among the IMS positive cell populations.

Our initial results showed that all RT-PCR positive cells were also EpCAM positive, indicating that the IMS procedure selected an epithelial cell population. However, only approximately 50% of all IMS-positive SLN samples expressed any of the four epithelial transcripts targeted by our RT-PCR assay. We then speculated that the EpCAM positive but epithelial transcript negative cells isolated from the SLNs could be tumor derived cells passing through an epithelial to mesenchymal transition (EMT). To investigate this we analyzed more IMS positive SLN samples using transcripts associated with EMT. This revealed that EpCAM+ and mammaglobin positive (hMAM+) cells represented a cell population also expressing other typical epithelial transcripts. In the EpCAM+ and hMAM negative cells these epithelial transcripts were lost or markedly reduced, while several mesenchymal markers were increased. The observed changes in gene expression were consistent with cells passing through EMT, indicating that this process could be important in the lymphatic dissemination of breast cancer.

## Materials and methods

### Patients and samples

The sentinel nodes used in this study were collected from January 2008 to March 2010. Patients with primary breast cancer and clinically negative axillary lymph nodes underwent sentinel lymph node biopsy (SLNB) and surgery at Ullevaal University Hospital. The majority of patients had T1 (≤2 cm) or T2 (> 2 cm ≤5 cm) tumors. In total, 120 SLN samples from as many patients have been analyzed in this study. The SLNB procedure was performed according to the guidelines of the Norwegian Breast Cancer Group [25]. Informed and written consent was obtained from all patients and the project was approved by the regional ethics committee. Sentinel lymph nodes from each of the patients were bisected along the longitudinal axis and one half was used for routine histopathological assessment. The other half was used for IMS and RT-PCR analysis.

### Immunomagnetic selection method

#### Antibodies and magnetic beads

The MOC31 (anti-EpCAM) monoclonal antibody (IQ Products, Groningen, The Netherlands) [26], and BM-7 (from Dr. S. Kaul, Heidelberg, Germany), reacting with the core protein of Mucin 1, were used in parallel for the detection and characterization of tumor cells in the disaggregated cell suspensions prepared from the sentinel nodes. Immunomagnetic M450 Dynabeads (Invitrogen, Oslo, Norway) with a diameter of 4.5 μm and coated with rat anti-mouse antibodies, were coated with the MOC31 or BM-7 antibodies as previously described [27].

#### Disaggregation of the sentinel lymph node tissue

The tissue from one half of the lymph node was placed in a petri dish containing 2 to 5 ml of PBS/1%HSA for disaggregation by the use of scalpels. A 70 μm cell strainer (BD Biosciences, Franklin Lakes, NJ, USA) was placed in a 50 ml tube and the fragmented tissue was filtered through the cell strainer into the tube. The cell strainer was washed with PBS/1%HSA. The filtered cell suspension was concentrated by centrifugation at 200 g for five minutes and the cells subsequently resuspended in 5 ml PBS/1%HSA and transferred to round bottom tubes for incubation with antibody-coated beads.

#### Immunomagnetic detection

Each sample was subjected to two different bead-antibody combinations, containing either MOC31 or BM-7-coated immunomagnetic beads, while uncoated beads were used for control. Beads were added to the cells in a ratio of 1:2, and the mixture was incubated under constant rotation for 30 minutes at 4°C in a volume of 1 ml. After incubation, the cells were diluted 1:3 with PBS/1%HSA and the tubes were placed in a magnet holder for selection of the cell-immunobead rosettes. Cells expressing the target antigen were trapped in the tube by the magnet, whereas the supernatant, containing unbound cells, was decanted and saved for RNA isolation. The enriched fraction, containing the viable rosetted cells, was resuspended in 200 μl PBS/1%HSA and placed on ice. A 20 μl sample of the positively enriched fraction was pipetted onto a slide and evaluated by microscopy using a Zeiss Axioscope (Carl Zeiss, Jena, Germany).

#### Criteria for positivity

A sample was defined as positive when it contained at least two cells, each with ≥ 5 immunomagnetic beads bound to its surface, simultaneously evaluating the size, morphology and three-dimensional picture seen in the microscope.

### RT-PCR analysis

#### RNA and cDNA synthesis

All RNA extractions were performed using Trizol Reagent (Invitrogen Life Science, Carlsbad, CA, USA). RNA concentration was routinely assessed on the NanoDrop 1000 instrument (Thermo Fisher Scientific, Waltham, MA, USA). Generally 1 μg of RNA was reverse transcribed using the qScript cDNA synthesis kit (Quanta BioSciences Inc., Gaithersburg, MD, USA) in a volume of 20 μl, then diluted to 50 μl using dH_2_O. The positive enriched fractions contain few cells and thus have low RNA counts. RNA from these fractions was not measured, and all available RNA was loaded into the 20 μl RT-synthesis reaction and diluted to 30 μl before PCR.

### Positive controls for RT-PCR

Mononuclear cells isolated from fresh bone marrow of a healthy donor were spiked with either SKBR3 or T47d cells in known ratios (1 SkBr3/10^4 ^MNC and 1 T47d/10^3 ^MNC). RNA was isolated using Trizol Reagent, dissolved in RNA storage solution (AM7000, Ambion/Applied Biosystems, Austin, TX, USA) and kept as frozen aliquots at -80°C. The final RNA preparation contained the equivalent of 100 SkBr3 cells/ug and 500 T47d cells/ug, and based on this each positive control PCR contained the equivalent of either 10 SkBr3 cells or 50 T47d cells. The T47d control was a positive control for AGR2 and TFF1 while the SkBR3 control was used for hMAM and SBEM. Controls were included on all PCR plates for quality assessment, and for relative quantification purposes. We used spiked samples and not pure cell lines to closer mimic the actual clinical samples.

#### Real-time PCR

Real-time PCR reactions were performed on the iCycler instrument from BioRad (Hercules, CA, USA). All reactions were run in parallel, and all samples were in 25 μl volume. Each primer mix contained 200 nM FAM-labeled probe, 300 nM of each primer and 1x Perfecta qPCR Supermix (Quanta BioSciences Inc.). Expression of YARS, a t-RNA synthetase, was used for sample validation and normalization of expression. The reference gene (*YARS*) had been tested in a panel of SLN samples and found to have equal expression per ng of cDNA. All primers have been validated using appropriate controls and negative and positive controls for all targets were always included in all PCR runs. Primers are designed using the probe finder software from Roche Applied Science available online at the Universal ProbeLibrary Assay Design Center [28], and all probes are from the Universal ProbeLibrary collection (Roche Applied Science). Primer sequences are listed in Additional file 1.

#### Criteria for positivity

The four markers hMAM, AGR2, TFF1 and SBEM were tested and found completely negative in normal bone marrow; therefore, no cut-offs were used on the cycle threshold (Ct) values. Only samples which showed positive signals from the reference gene YARS in both parallels were considered successfully analyzed. To count as a positive sample at least one other marker (either hMAM; AGR2, TFF1 or SBEM) in at least one cell fraction would have to be positive in both parallels.

### EMT RT-PCR arrays

Since our RT-PCR results showed no differences between EpCAM-positive and Mucin 1 positive cells, we only used EpCAM positive and EpCAM depleted cell fractions in the array tests. RNA from EpCAM positive SLN fractions were first analyzed for YARS and hMAM expression using our own primers. All RNA was DNase-treated (Ambion, Austin, TX, USA). Samples denoted by numbers (for example, 540+ and 540-) represent EpCAM positive and depleted cells from single SLN samples. These were analyzed using approximately 500 ng DNase treated RNA per array. In order to obtain a higher amount DNase treated RNA before cDNA synthesis, some samples were pooled according to hMAM expression (details of samples are presented in the supplementary data). These are denoted by letters, for example, G+, L+, A+, Q+, P+, K- and C- on the arrays, and were analyzed using 2.5 μg DNase treated RNA per array. Both quantities are within the range recommended by the manufacturer. The cDNA was mixed with Perfecta SYBRGreen supermix (Quanta BioSciences Inc.) and applied on to ready bought 96-well plates containing primers for EMT-associated genes (SABiosciences, Frederick, MD, USA). The samples were run on the iCycler instrument from BioRad using the recommended protocol.

### Data analysis

All expression data were analyzed using the software GenEx (MultiD analyses AB, Gothenburg, Sweden). The software includes an application (Normfinder), which determines the best reference gene(s) for normalization of expression data. The arrays contained five possible reference genes, ACTB, GAPDH, B2M, HPRT1 or RPL13A, and Normfinder determined ACTB and RPL13A to be the best references in our dataset. We also tried alternative normalization strategies, but this did not alter the results. The relative expression of target genes was calculated by comparing all samples against the average expression of each gene (Mean log expression = 1). This is an application included in the GenEx software.

### Statistical analysis

Statistical analysis and hierarchical clustering was also performed using the GenEx software. Two-tailed unpaired t-tests were used to compare array results obtained from all EpCAM+ fractions, EpCAM+/hMAM+ or EpCAM+/hMAM- fractions to results obtained from EpCAM depleted cell fractions. When genes failed the Kolomogorov-Smirnov test (used to determine if samples show a normal distribution) a non-parametric Mann-Whitney test was used to calculate *P*-values. A *P*-value ≤0.05 was considered statistically significant.

### Validation of array-results

Real-time RT-PCR for EpCAM, EGFR, Wnt5b, SPARC, Col1A2, MMP2, ERRB2, hMAM and YARS were performed as previously described for other RT-PCR targets, but due to low amount of template all reactions were run as singles. YARS was used for normalization and fold change was calculated relative to the average expression of each gene. All calculations and statistics were performed using the GenEx software. Primer sequences are listed in Additional file 1

## Results

### hMAM, AGR2, TFF1 and SBEM were selected for RT-PCR studies

For detection of breast cancer cells in lymph nodes we searched the literature for transcripts reported to be either breast or epithelial associated. We examined the expression of several potential markers in a panel of 18 normal bone marrow samples, 6 breast cancer cell lines and 8 cell lines of other human malignancies. The four genes hMAM (mammaglobin, MGB1, SCGB2A2) [29], AGR2 (anterior gradient homolog 2) [30], TFF1 (trefoil factor 1, pS2) [31] and SBEM (small breast epithelial mucin) [32] were all found negative in bone marrow, indicating their absence in normal hematopoietic tissue. hMAM proved to be the most breast specific marker, although its expression was restricted to only four of six breast cancer cell lines. TFF1, AGR2 and SBEM were expressed to a varying degree in all breast cancer cell lines, but also in other malignancies (Table 1).

**Table 1:** Expression of hMAM, AGR2, TFF1 and SBEM measured by RT-PCR

Cell line	hMAM	AGR2	TFF1	SBEM
T47d	+	+	+	+
PM1	+	+	+	+
SKBR-3	+	+	+	+
MCF7	+	+	+	+
MDA-MB231	-	+	+	+
MA11	-	+	+	+
FEM-XI	+	-	-	-
SKMEL 28	-	-	-	+
HTB-182	+	+	+	+
786-O	-	+	+	-
D54MG	-	+	-	-
HT-29	-	+	+	-
T84	-	+	+	-
WiDr	-	+	+	-
HuFib	-	-	-	-
18 Norm. BM	-	-	-	-

Expression of the four epithelial transcripts hMAM, AGR2, TFF1 and SBEM was evaluated in selected cell lines and healthy bone marrow by RT-PCR.

### Comparison of IMS and RT-PCR results

**Figure 1:**
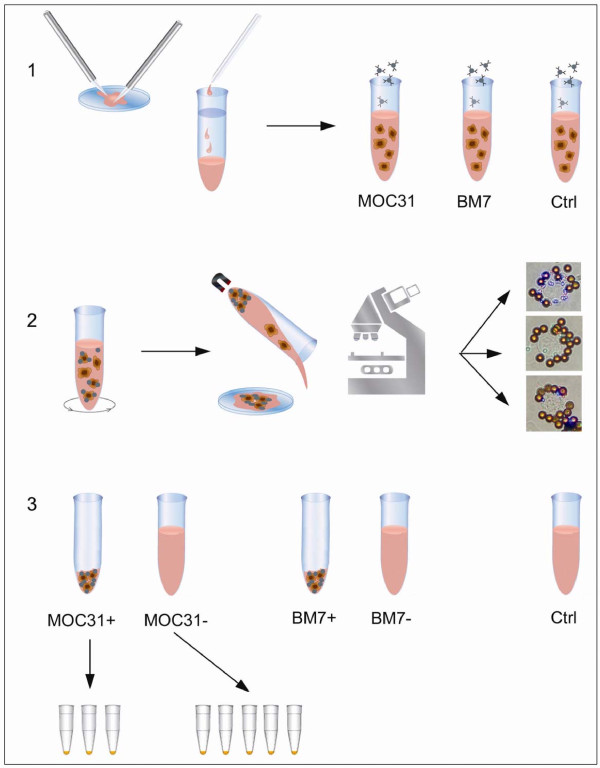
**Schematic overview of the combined IMS and RT-PCR analysis**. 1) Fresh lymph nodes were disintegrated in sterile PBS. The resulting cell suspension was filtered, centrifuged, diluted in PBS and aliquoted. The cells were then mixed with either MOC31 coated magnetic beads (anti-EpCAM), BM-7 coated magnetic beads (anti-Mucin 1) or beads without antibodies for control. **2**) The cells and beads were incubated at 4°C for 30 minutes, and placed on a magnet for cell separation. Cells positive for either MOC31 or BM-7 were retained by the magnet while the negative cells were decanted off into separate tubes. **3**) All cell fractions were subjected to individual RNA isolation and RT-PCR analysis. One patient sample could thus yield a maximum of five different cell fractions for RT-PCR. IMS positive fractions were analyzed for three PCR targets while the much larger negative fractions were analyzed using five targets.

To elucidate the sensitivity and specificity of the IMS method we performed, in sequence, IMS screening and RT-PCR analysis of clinical SLN samples. The IMS assay employed two antibodies against cell surface markers, MOC31 (anti-EpCAM) and BM-7 (anti-Mucin 1) for cell separation. Both immunomagnetically selected cells and their corresponding depleted fractions, that is, the remaining cell population after positive selection, were collected and subjected to RT-PCR using the four selected transcripts as molecular markers. The control fraction, corresponding to the original, non-separated cell population, was also analyzed. A schematic view of the IMS and RT-PCR procedure is presented in Figure 1.

Each lymph node sample gave a maximum of five cell fractions for RT-PCR, and from a total of 71 patients we successfully obtained both IMS and RT-PCR data.

All 71 SLNs were IMS positive with the MOC31 antibody, and 53 of these were also positive with BM-7. To check for associations between IMS and RT-PCR results, we compared the RT-PCR findings with the IMS data (Table 2).

**Table 2:** Comparison of IMS and RT-PCR results

IMS results	RT-PCR +	RT-PCR ÷	Total
MOC31 +	35 (49%)	36 (51%)	71
MOC31 ÷	-	-	-
BM7 +	27 (51%)	26 (49%)	53
BM7 ÷	8 (44%)	10 (56%)	18

The RT-PCR results (positive/negative) are shown as number (%) of the total samples that were positive or negative with each of the antibodies used in the immunomagnetic selection (IMS).

Surprisingly, the RT-PCR positive and negative samples were evenly distributed regardless of which surface marker they were related to. Of the 71 sentinel lymph nodes with EpCAM positive cells, 35 were RT-PCR positive and 36 were negative. Similarly, of 53 Mucin 1 positive samples 27 (51%) were RT-PCR positive.

We then compared the number of IMS positive cells (based on microscopy inspection of 1/10 of the sample) detected in each sample to the RT-PCR results (Table 3). For simplicity, only results obtained from the MOC31 IMS were used. The numbers varied greatly from only four positive rosettes in one aliquot to > 500 positive rosettes in one aliquot. However, the 35 RT-PCR positive samples and the 36 RT-PCR negative samples did not show any difference with respect to the number of IMS-positive cells detected (*P *= 0.48).

**Table 3:** EpCAM positive cells in RT-PCR positive and RT-PCR negative samples

RT-PCR positive samples			RT-PCR negative samples		
Lowest # of IMS+ cells	Highest # of IMS+ cells	Mean # IMS+/sample	Lowest # of IMS+ cells	Highest # IMS+ cells	Mean # IMS+/sample
4	> 500	97	3	245	79

The number of cells positive by immunomagnetic selection (IMS) in one-tenth of the SLN sample is shown in comparison to the final RT-PCR result.

### EpCAM and Mucin 1 immunomagnetic selection enriched for RT-PCR marker expressing cells

**Figure 2:**
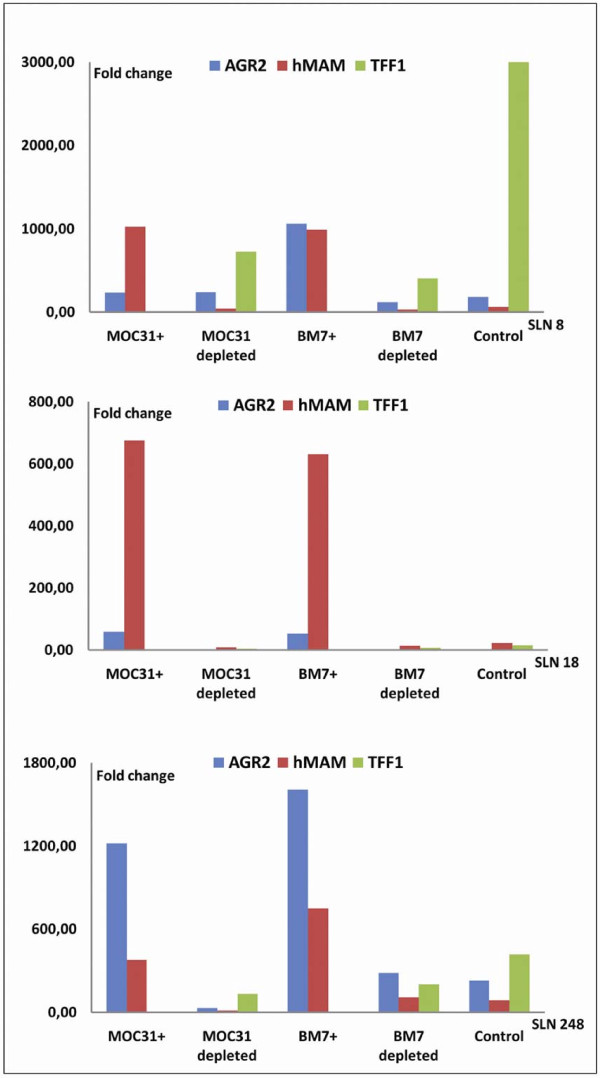
**EpCAM and Mucin 1 enrichment also enriched for cells expressing epithelial transcripts**. Cells from SLN samples were separated by IMS and the resulting cell fractions were analyzed by RT-PCR. Examples from three different RT-PCR positive SLN samples are shown. In all graphs, the first fraction corresponds to the MOC31 (anti-EpCAM) enriched cell population, that is, all cells which expressed the surface antigen EpCAM. The next fraction is the MOC31 depleted cells, that is, cells which did not express EpCAM. These are followed by Mucin 1 expressing cells (BM-7 positive), Mucin 1-depleted cells, and finally the control fraction corresponding to the non-separated cell population of the SLN sample. Due to the low amounts of template, all IMS enriched fractions were only analyzed for two PCR targets, while depleted- and control fractions were analyzed using all targets. SBEM was analyzed separately, and was positive in all three samples (not shown). The reference gene YARS was used for normalization of RT-PCR values and fold change was calculated relative to a positive control included on all PCR plates.

To further examine the relationship between IMS and RT-PCR positivity we performed a comparison of RNA marker expression in the different IMS-defined cell fractions. In 15 of the 35 RT-PCR positive SLN samples all five available cell fractions were analyzed by PCR, and in these the highest RT-PCR marker expression levels was found in the EpCAM and Mucin 1 positively selected fractions. The remaining cell populations were clearly depleted of RT-PCR marker expressing cells, proving that the IMS did select cells with an epithelial expression profile. RT-PCR signals from Mucin 1+ or EpCAM+ cells isolated from the same SLN were similar in all samples, indicating that the two immunomagnetically selected cell fractions were identical with respect to expression of the four chosen RNA markers. Results from representative patient samples are shown in Figure 2.

### hMAM, AGR2, TFF1 and SBEM were expressed by the same cell population

Of the 35 RT-PCR positive samples, as many as 31 (88%) showed hMAM expression, making it the most frequently expressed marker. In five cases hMAM was the only transcript detected. Of the four hMAM negative samples three were positive for AGR2 and either TFF1, SBEM or both, whereas the fourth was only positive for SBEM. A total of 34 of the 35 (97%) positive samples would have been identified using only hMAM and AGR2. The data demonstrate that our RT-PCR markers closely identified the same cell population.

### A population of EpCAM positive cells identified in the lymph nodes lack typical epithelial transcripts

Approximately 50% of the IMS-positive samples showed no expression of the breast/epithelial specific transcripts, regardless of the number of IMS positive cells detected in the samples (Tables 2 and 3). This discrepancy might be explained if the antibodies employed for IMS bind also to subpopulations of normal cells in the SLN, or alternatively if a large group of tumor cells do not express the epithelial RT-PCR markers. As shown in Figure 2 the immunomagnetic selection of EpCAM or Mucin 1 positive cells enriched for RT-PCR transcript expressing cells, demonstrating that all RT-PCR positive cells were EpCAM positive, but not vice versa. To further investigate this we extended the RT-PCR studies by the use of pathway-focused PCR-array plates, and tested for transcripts associated with the process of epithelial to mesenchymal transition. EMT is naturally occurring during embryonic development, and is believed to be reactivated in carcinoma metastasis [33-36]. Epithelial cells undergoing EMT down regulate typical epithelial markers and become mesenchymal-appearing cells with an increased capability for motility and invasion of surrounding tissues. If a similar process occurs in breast cancer cells that migrate to lymph nodes this would have affected the expression of epithelial transcripts including those we used in our RT-PCR assay.

**Figure 3:**
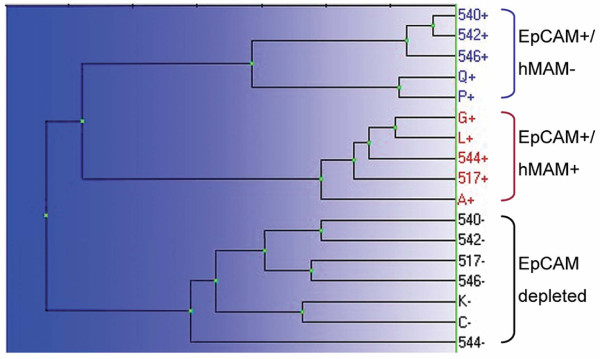
**Hierarchical clustering of RT-PCR array data separates cells isolated from SLNs into three groups** . cDNA from a total of 17 different samples, representing both EpCAM positive and EpCAM depleted cell fractions, were analyzed on RT-PCR arrays targeting transcripts associated with EMT. Expression data from the arrays were analyzed using the software GenEX. The hierarchical clustering analysis separated the samples into three groups: EpCAM+/hMAM+ cells, EpCAM+/hMAM- cells and EpCAM depleted cells.

A total of 17 IMS isolated samples representing EpCAM positive or EpCAM depleted cell fractions from SLNs were successfully analyzed on 96-well PCR-array plates. Prior to array analysis we scored all samples for hMAM expression. Information regarding the number of bead-rosetted cells and hMAM status of each sample is presented in the Additional file 2. To further examine how the patient samples differed based on expression of the EMT associated genes we first performed an unsupervised hierarchical clustering analysis of the normalized expression data (Figure 3). This divided all samples into two major groups, the EpCAM positive cells (10 arrays) and the EpCAM depleted cells (7 arrays). The group consisting of EpCAM positive cells was further split into two clusters, one containing the EpCAM+/hMAM- cells (five arrays) and one containing the EpCAM+/hMAM+ cells (five arrays).

We then performed statistical t-tests on array results obtained from the different groups, that is, we compared EpCAM+/hMAM+ (5 arrays) vs EpCAM+/hMAM- (5 arrays), EpCAM+ (10 arrays) vs EpCAM depleted cells (7 arrays), and EpCAM+/hMAM- (5 arrays) vs EpCAM depleted (7 arrays). The low number of samples present in each group reduces the power of statistical testing, but the resulting gene lists are useful for evaluating trends in the data. All results from the t-tests are available in Additional file 2.

### Epithelial transcripts are associated with hMAM expressing cells

**Figure 4:**
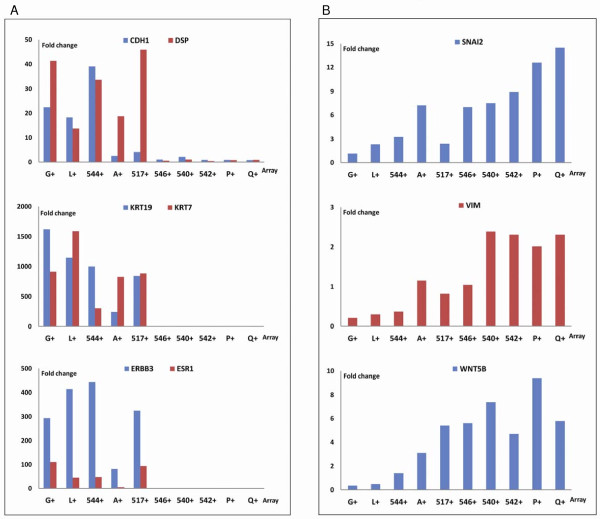
**Expression results from 10 EMT arrays representing only EpCAM+ cell populations**. The first five samples (G+, L+; 544+, A+ and 517+) also expressed hMAM while the last five (546+, 540+, 542+, P+ and Q+) were negative for hMAM as determined by RT-PCR. Fold changes were calculated relative to the average expression of each gene. **a**) All hMAM expressing cell populations co-expressed other epithelial transcripts as E-cadherin (CDH1), desmoplakin (DSP), and cytokeratins 7 and 19 (KRT7/19). The estrogen receptor (ESR1) and ERBB3 (HER3) were also expressed mainly by hMAM positive cells. **b**) In contrast, EpCAM+/hMAM- cells show increased expression of EMT markers; the E-cadherin repressor Snai2, the mesenchymal filament vimentin (Vim) and Wnt5B, a member of the wnt-signaling pathway, were all increased in EpCAM+/hMAM- compared with EpCAM+/hMAM+ cell populations.

When comparing EpCAM+/hMAM+ cells with EpCAM+/hMAM- cells the array results revealed that cells expressing mammaglobin also expressed high levels of other known epithelial markers such as E-cadherin (CDH1), epithelial cytokeratins (KRT19 and KRT7), and desmoplakin (DSP). This confirmed the epithelial nature of these EpCAM+/hMAM+ cells, and was in agreement with our initial findings from the RT-PCR-assay where the use of four epithelial markers gave little additional information compared to the use of hMAM alone. All epithelial transcripts seem to be expressed by the same population of EpCAM+ cells. The estrogen receptor (ESR1) and ERBB3 (a member of the ErbB family) were also significantly higher expressed in EpCAM+/hMAM+ compared with EpCAM+/hMAM- cells (Figure 4a). In contrast, the EpCAM+/hMAM- cells showed a marked decrease in E-cadherin expression, and no expression of KRT7 or KRT19, but an increased expression of the transcription factor Snai2 (Slug), a known repressor of E-cadherin, the mesenchymal filament vimentin and Wnt 5B, a component of the Wnt signaling pathway (Figure 4b).

EMT is characterized by a down-regulation in epithelial markers, particularly E-cadherin, and an up-regulation of mesenchymal markers, particularly vimentin. These findings are thus consistent with the EpCAM+/hMAM- cells being in an EMT-state.

### Transcripts associated with EpCAM enrichment

**Figure 5:**
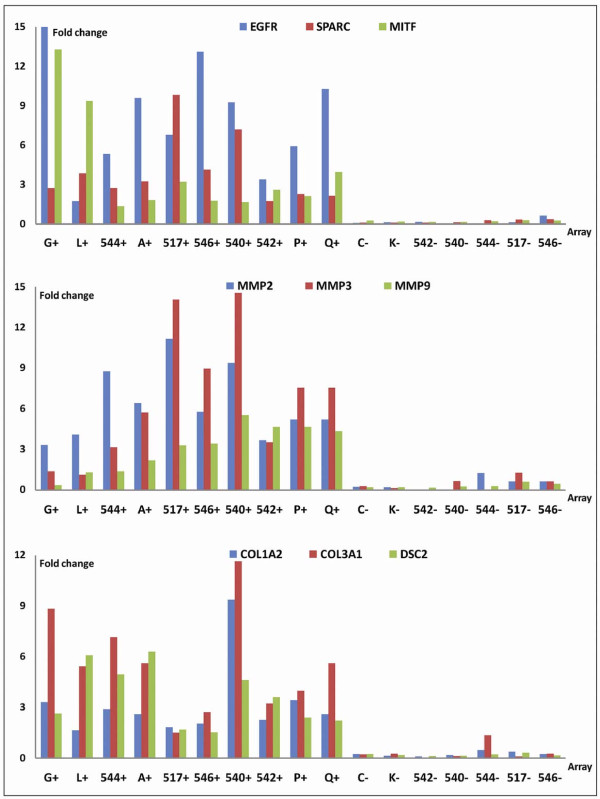
**EpCAM enrichment by IMS enriched for cells expressing transcripts associated with EMT**. All graphs show expression results from 17 EMT arrays representing EpCAM+/hMAM+ cells (first five arrays), EpCAM+/hMAM- cells (next five arrays) and EpCAM depleted cells (last seven arrays). Several transcripts were significantly higher expressed in all EpCAM+ fractions compared with the EpCAM depleted cells. This applied to EGFR, MITF and SPARC (upper panel), members of the metalloproteinase family (middle panel), collagens Col1A2 and Col3A1 and desmocollin (DSC2, lower panel). Note: sample G+ expressed more EGFR (actual value 419), but this is shortened to fit in the graph. Fold change is calculated relative to the average expression of each gene.

A number of targets present on the EMT array showed significantly increased expression in all EpCAM enriched cell populations compared with the EpCAM depleted cell fractions. This applied to SPARC, an indirect repressor of E-cadherin implicated in cellular invasion and motility (for a recent review see [37]), the transcription factor MITF, and the epidermal growth factor EGFR. EGFR belongs to the ErbB family of receptor tyrosine kinases (which also includes ERBB2 and ERBB3) and members of this family have been implicated both in EMT induction, and in tumor progression. Members of the family of metalloproteinases (MMP2/3/9), which are matrix degrading enzymes, collagens (Col1A2, Col3A1) and desmocollin (DSC2), a protein important for the formation of desmosomes and primarily found in epithelial cells, were also enriched for in all EpCAM positive cell fractions (Figure 5).

That so many genes were co-expressed with EpCAM, regardless of hMAM status, confirms that the EpCAM+ cells represented a distinct cell population, different from the EpCAM depleted cells.

### Validation of array-results

To validate the results from the RT-PCR array analysis we selected some genes identified as "EpCAM associated" and designed our own RT-PCR assays specific for EGFR, Wnt5b, SPARC, MMP2, and Col1A2. RT-PCR primers for EpCAM, ERBB2, and hMAM were also included in this panel. The selected assays were used to analyze 17 new SLN samples, all sorted by IMS into EpCAM enriched and EpCAM depleted cell populations.

**Figure 6:**
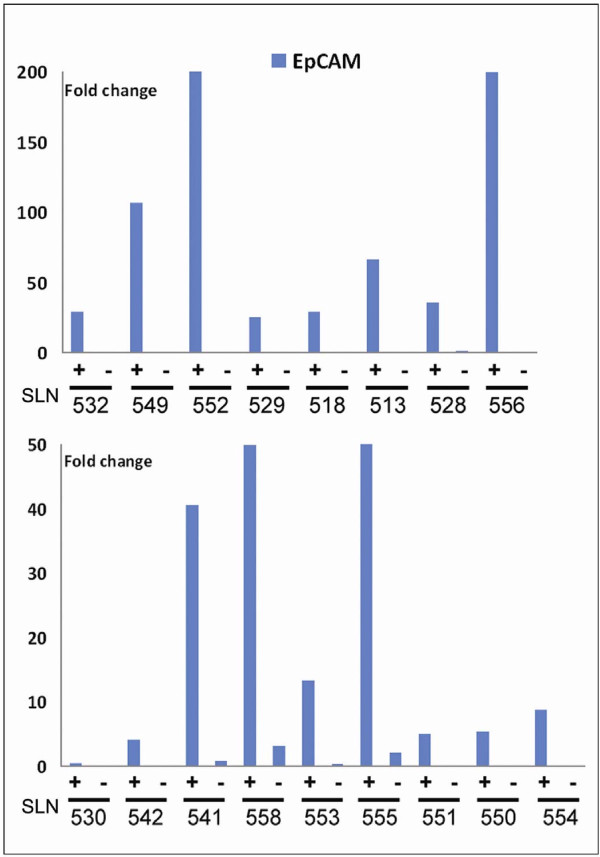
**EpCAM-IMS enriched for EpCAM RNA expressing cells**. EpCAM expression was significantly increased in EpCAM-IMS enriched cell populations compared with the corresponding EpCAM depleted cell fraction. This applied to both EpCAM+/hMAM+ cell populations (upper panel) and to EpCAM+/hMAM- cell populations (lower panel). Note that the scale is different, reflecting the generally lower expression level of EpCAM in EpCAM+/hMAM- cells compared with the EpCAM+/hMAM+ populations. To fit the graphs the value for sample 552+ has been set to 200 (actual value 605), while 555+ has been set to 50 (actual value 70). Fold change is calculated relative to the average expression of EpCAM in all samples.

**Figure 7:**
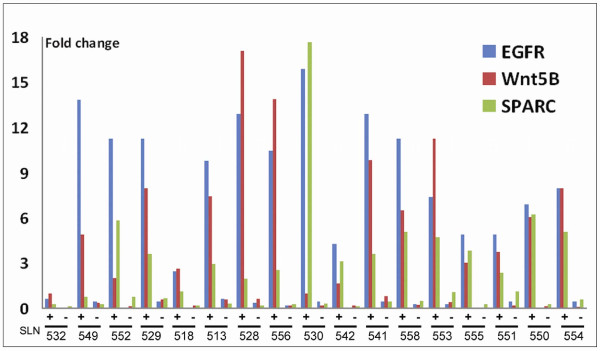
**EpCAM-IMS enriched cell populations are also enriched for EGFR, Wnt5B and SPARC RNA expression** . RT-PCR analysis targeting EGFR, Wnt5B and SPARC all showed increased expression of these transcripts in EpCAM enriched cell populations compared with the corresponding EpCAM depleted cell populations (separated by IMS). This was true in SLN samples with hMAM expression (first eight samples; SLN 532 to 556) and in SLN samples negative for hMAM (last nine samples, SLN 530 to 554). Fold change was calculated relative to the average expression of each gene.

All the selected transcripts showed significantly higher expression (*P *≤0.05) in the EpCAM enriched cell populations compared with the EpCAM depleted cells, thereby confirming the array-data. This also applied to EpCAM itself, as measured by RT-PCR. However, EpCAM expression was markedly higher in the eight samples also positive for mammaglobin (EpCAM +/hMAM+) than in the nine samples not expressing hMAM (Figure 6). In a t-test, EpCAM was the gene with the single largest mean difference between groups, both when all EpCAM+ and EpCAM depleted cells were compared (difference 9.28), and also when comparing EpCAM+/hMAM+ (difference 10.78) or EpCAM+/hMAM- (difference 7.94) to the EpCAM depleted cells. This confirms that the IMS-enrichment using MOC31 targets EpCAM-RNA expressing cells. EGFR, SPARC and Wnt5B were the genes showing the largest difference between EpCAM+ and EpCAM depleted cells. SPARC was slightly higher in the EpCAM+/hMAM- cells, while Wnt5B and EGFR were evenly expressed by all EpCAM+ cell populations (Figure 7).

## Discussion

In early stage breast cancer it is well established that the metastatic status of the SLN is an important prognostic parameter, but an optimal method of SLN analysis is still not available. In this study we sought to compare two sensitive methods that both seek to identify tumor cells in sentinel lymph nodes, immunomagnetic selection (IMS) using antibodies targeting the epithelial surface proteins EpCAM and Mucin 1, and RT-PCR targeting breast-epithelial transcripts. The objective was to examine to what extent the results obtained with the different methods did overlap and if one method was better than the other. Our results showed that all RT-PCR positive cells were EpCAM positive, but the EpCAM-IMS assay identified twice as many positive specimens as RT-PCR targeting epithelial transcripts.

When we investigated gene expression using other RT-PCR markers we found expression profiles consistent with the EpCAM positive but epithelial transcript negative cells (EpCAM+/hMAM-) being tumor derived cells passing through an epithelial to mesenchymal transition (EMT). EMT is a developmental program used in cell differentiation during embryogenesis, but parts of this genetic program is believed to be reactivated during metastasis in order to transform malignant epithelial cells into motile and invasive mesenchymal-like cells [33,34,38]. EMT initiates changes in gene transcription which allows epithelial cells to lose polarity and cell-cell contact, detach from the epithelial cell layer, modify the extracellular matrix and migrate through tissues. It is believed that EMT is a transient state and that the process is reversed once the cells form a metastatic lesion. However, as EMT or EMT-like mechanisms affect gene expression and protein translation, identifying metastatic cells while they are *en route *to a secondary organ might require different biological markers than the markers used to identify cells in the primary tumors.

The challenge in micrometastasis detection consists of identifying very few tumor cells in a background of normal cells. However, tumor cells that pass through EMT adopt the phenotypic features of mesenchymal tissue, thereby making them difficult to distinguish from the surrounding stroma. Lymph nodes are home to a variety of different cell populations, many of which have a mesenchymal character. Consequently, clear evidence of oncogenic EMT is hard to prove in samples of lymph node tissue. In addition, genes are rarely implicated in only one cellular process; their effects may be quite different depending on the context in which they are expressed. Thus, we cannot at present be sure whether we are actually observing cancer cells passing through EMT or cells involved in other differentiation processes. However, a number of indications point to these cells being tumor derived. First, all cells were selected by EpCAM protein expression on the cell surface. The efficiency of this enrichment is demonstrated by EpCAM RT-PCR results from MOC31 enriched and depleted cell fractions (Figure 6). EpCAM is a cell surface protein generally used as an epithelial specific antigen. Second, our EpCAM-IMS assay proved to enrich for epithelial-transcript expressing cells as demonstrated both by our initial RT-PCR assay targeting four epithelial transcripts (Figure 2) and the array results (Figure 4a). Third, all EpCAM positive cells consistently showed increased expression of a number of transcripts compared with the EpCAM depleted cells, demonstrating that EpCAM positive cells represent a distinct cell population independent of hMAM expression. Some genes were nearly exclusively expressed by EpCAM positive cells (EGFR, Serpine-1 (PAI-1), Snai2, Col3A1 and MMP2/3), while others were enriched for (SPARC, MITF, MMP9, MST1R Col1A2/Col5A2, Wnt5A/Wnt5B and others). This expression pattern was confirmed for some selected genes using different RT-PCR primers and a new set of SLN samples (Figure 7). Fourth, the changes in expression observed between EpCAM+/hMAM+ and EpCAM+/hMAM- cell populations were consistent with changes expected to occur during EMT (Figure 4a, b). Thus, as expression of E-cadherin and other epithelial transcripts were lost or greatly reduced, mesenchymal markers as Snai2 (Slug), vimentin, Wnt5B, and MMP9 were increased. Collectively these results indicate that EpCAM+/hMAM- cells are tumor-derived cells similar to EpCAM+/hMAM+ cells, but with phenotypic alterations compatible with an EMT state.

Epithelial cytokeratins are commonly used for antibody-based detection of tumor deposits in SLN by immunohistochemistry (IHC). Recently, both hMAM and CK19 also received FDA approval as markers for the RT-PCR based analysis of SLNs in a diagnostic kit (GeneSearch Breast Lymph Node (BLN) assay; Veridex LLC, Raritan, NJ, USA). Studies that have compared RT-PCR and IHC on SLN samples generally find that the methods agree [39-41], demonstrating that epithelial protein- and RNA- markers are expressed by the same cells. As shown in Figure 4a our EpCAM+/hMAM+ cells also expressed CK19, along with other epithelial markers. However, hMAM is not a universal marker of breast cancer derived tumor cells, as demonstrated by our initial test where only four of six breast cancer cell lines were found to express hMAM. Similarly, the expression of different cytokeratins has been found to vary considerably in DTC detected in bone marrow from breast cancer patients [42]. As our EpCAM IMS assay detected both hMAM +/- cells and CK +/- cells, this demonstrates that EpCAM is able to capture a broader range of tumor cells. Importantly, these mesenchymal-like cells will remain undetected when purely epithelial markers are used.

All patients included in this study were early stage, clinically node-negative patients. Statistically, the expected five-year survival rate is between 85 and 95% (based on T1/T2 patients, prognoses from [43]). Our EpCAM IMS assay therefore detects cells in SLNs of far more patients than those who can be expected to suffer from relapse later. However, although these cells have migrated to the lymph node we do not know whether they will spread further from there. Currently, it is not known which characteristics that identify metastatic founder cells or which factors that influence the fate of cells disseminated to lymph nodes. Emerging evidence from studies on bone marrow suggests that tumor cell dissemination is a common event which starts at an early stage, and that disseminated cells may evolve at the ectopic site independently of the primary tumor [44,45]. We do not know what potential cells disseminated to lymph nodes have to give rise to distant metastases, but the data are relevant in the context of understanding tumor cell dissemination and behavior.

Antibodies targeting EpCAM are commonly used for detecting metastatic cells in blood or bone marrow [46-48]. Notably, EpCAM is approved for diagnostic use in the CellSearch assay which aims to detect circulating breast cancer cells [49-51]. It has been suggested that EpCAM might be down-regulated as a consequence of EMT [52,53]. However, our data indicate the concomitant expression of EpCAM and EMT markers on metastatic cells in SLNs, in line with recent results obtained on samples from bone marrow [54] and blood [55]

Our EpCAM RT-PCR assay consistently showed higher EpCAM expression in EpCAM+/hMAM+ samples than in EpCAM+/hMAM- samples (Figure 6). This difference did not reflect results obtained in the EpCAM IMS assay where many samples later found to be hMAM negative contained high numbers (> 1,000) of heavily MOC31-rosetted cells. This apparent imbalance in EpCAM protein expression and RNA expression could reflect the dynamics of an EMT process, where the halt of mRNA transcription and degradation of mRNA transcripts might be more rapidly executed than the internalization and degradation of proteins already present in the cell membrane. Thus, in cells undergoing EMT, proteins associated with an epithelial profile could be detectable longer than the transcripts encoding them.

Breast cancer is a very heterogeneous disease with variation both on the DNA, RNA and protein levels [56,57]. This heterogeneity is found within tumors as well as between patients, and can be explained by several mechanisms; the origin of the cell causing the cancer [58], the different environmental factors involved in driving the tumor, and transient changes in cellular status associated with malignant progression, such as EMT [33-35,38,59]. We observed a large heterogeneity on the RNA level among our EpCAM enriched cell populations, also in hMAM expression. Since our RT-PCR results are based on the pool of EpCAM positive cells, it can not be derived from this whether the variable hMAM intensity was due to a difference between patients, or if patients with low hMAM expression had a mixture of EpCAM+/hMAM+ and EpCAM+/hMAM- cells. EMT is a plastic process and the transition from an epithelial cell to a mesenchymal-like cell is probably a continuum rather than an abrupt change. Thus, it will be interesting to analyze EpCAM positive cells at the single cell level to see if such differences in cell state are detectable within individual patients.

## Conclusions

Our analysis of SLN samples indicates that the activation of EMT like processes is important in the lymphatic dissemination of breast cancer. EpCAM thus appear to be a more universal marker of breast cancer micrometastasis than pure epithelial derived targets, as it will also detect tumor cells with EMT-like characteristics.

## Abbreviations

AGR2: anterior gradient homolog 2; BLN: breast lymph node; Ct: cycle threshold; EMT: epithelial to mesenchymal transition; EpCAM: epithelial cell adhesion molecule; hMAM: human mammaglobin; IHC: immunohistochemistry; IMS: immunomagnetic separation; RT-PCR: reverse-transcription polymerase chain reaction; SLN: sentinel lymph node; SLNB: sentinel lymph node biopsy; TFF1: Treefoil factor 1; YARS: tyrosyl-tRNA synthetase

## Competing interests

The authors declare that they have no competing interests.

## Authors' contributions

ST planned and performed all experiments. RK arranged the collection of clinical material. ST, KA and OF participated in the design of the study, discussion and interpretation of results. ST drafted the manuscript and OF revised it. All authors read and approved the final manuscript.

## Supplementary Material

Additional file 1 **Primers and probes used for RT-PCR**. Sequence of primers designed using ProbeFinder from Roche Applied Science and the corresponding probes from the Universal ProbeLibrary [28] Click here for file

Additional file 2 **SLN samples used on the EMT-arrays and EMT RT-PCR with calculations**. A complete overview of all clinical samples used in this study. The number of Immunomagnetic selected (IMS) positive cells per sample and the hMAM status as determined by RT-PCR is listed. Then the EMT-array RT-PCR data are shown, both raw Ct values and the normalized expression data. Following this is the hierarchical clustering analysis and the different t-tests. Click here for file

## References

[B1] Carter CL Allen C Henson DE Relation of tumor size, lymph node status, and survival in 24,740 breast cancer cases Cancer 1989 63 181 187 10.1002/1097-0142(19890101)63:1<181::AID-CNCR2820630129>3.0.CO;2-H 2910416

[B2] Fisher B Bauer M Wickerham DL Redmond CK Fisher ER Cruz AB Foster R Gardner B Lerner H Margolese R Relation of number of positive axillary nodes to the prognosis of patients with primary breast cancer. An NSABP update Cancer 1983 52 1551 1557 10.1002/1097-0142(19831101)52:9<1551::AID-CNCR2820520902>3.0.CO;2-3 6352003

[B3] Fitzgibbons PL Page DL Weaver D Thor AD Allred DC Clark GM Ruby SG O'Malley F Simpson JF Connolly JL Hayes DF Edge SB Lichter A Schnitt SJ Prognostic factors in breast cancer. College of American Pathologists Consensus Statement 1999 Arch Pathol Lab Med 2000 124 966 978 10888772 10.5858/2000-124-0966-PFIBC

[B4] Krag D Weaver D Ashikaga T Moffat F Klimberg VS Shriver C Feldman S Kusminsky R Gadd M Kuhn J Harlow S Beitsch P The sentinel node in breast cancer--a multicenter validation study N Engl J Med 1998 339 941 946 10.1056/NEJM199810013391401 9753708

[B5] Giuliano AE Jones RC Brennan M Statman R Sentinel lymphadenectomy in breast cancer J Clin Oncol 1997 15 2345 2350 9196149 10.1200/JCO.1997.15.6.2345

[B6] Cote RJ Peterson HF Chaiwun B Gelber RD Goldhirsch A Castiglione-Gertsch M Gusterson B Neville AM Role of immunohistochemical detection of lymph-node metastases in management of breast cancer. International Breast Cancer Study Group Lancet 1999 354 896 900 10.1016/S0140-6736(98)11104-2 10489948

[B7] Cserni G Metastases in axillary sentinel lymph nodes in breast cancer as detected by intensive histopathological work up J Clin Pathol 1999 52 922 924 10.1136/jcp.52.12.922 10711258 PMC501663

[B8] Groen RS Oosterhuis AW Boers JE Pathologic examination of sentinel lymph nodes in breast cancer by a single haematoxylin-eosin slide versus serial sectioning and immunocytokeratin staining: clinical implications Breast Cancer Res Treat 2007 105 1 5 17221160 10.1007/s10549-007-9728-z PMC2001221

[B9] Motomura K Komoike Y Inaji H Hasegawa Y Kasugai T Noguchi S Koyama H Multiple sectioning and immunohistochemical staining of sentinel nodes in patients with breast cancer Br J Surg 2002 89 1032 1034 10.1046/j.1365-2168.2002.02177.x 12153631

[B10] de Boer M van Deurzen CH van Dijck JA Borm GF van Diest PJ Adang EM Nortier JW Rutgers EJ Seynaeve C Menke-Pluymers MB Bult P Tjan-Heijnen VC Micrometastases or isolated tumor cells and the outcome of breast cancer New Engl J Med 2009 361 653 663 10.1056/NEJMoa0904832 19675329

[B11] Reed J Rosman M Verbanac KM Mannie A Cheng Z Tafra L Prognostic implications of isolated tumor cells and micrometastases in sentinel nodes of patients with invasive breast cancer: 10-year analysis of patients enrolled in the prospective East Carolina University/Anne Arundel Medical Center Sentinel Node Multicenter Study J Am Coll Surg 2009 208 333 340 10.1016/j.jamcollsurg.2008.10.036 19317993

[B12] Tan LK Giri D Hummer AJ Panageas KS Brogi E Norton L Hudis C Borgen PI Cody HS Occult axillary node metastases in breast cancer are prognostically significant: results in 368 node-negative patients with 20-year follow-up J Clin Oncol 2008 26 1803 1809 10.1200/JCO.2007.12.6425 18332473

[B13] Querzoli P Pedriali M Rinaldi R Lombardi AR Biganzoli E Boracchi P Ferretti S Frasson C Zanella C Ghisellini S Ambrogi F Antolini L Piantelli M Iacobelli S Marubini E Alberti S Nenci I Axillary lymph node nanometastases are prognostic factors for disease-free survival and metastatic relapse in breast cancer patients Clin Cancer Res 2006 12 6696 6701 10.1158/1078-0432.CCR-06-0569 17121888

[B14] Brunsvig PF Flatmark K Aamdal S Hoifodt H Le H Jakobsen E Sandstad B Fodstad O Bone marrow micrometastases in advanced stage non-small cell lung carcinoma patients Lung Cancer (Amsterdam, Netherlands) 2008 61 170 176 10.1016/j.lungcan.2007.12.018 18261824

[B15] Faye RS Aamdal S Hoifodt HK Jacobsen E Holstad L Skovlund E Fodstad O Immunomagnetic detection and clinical significance of micrometastatic tumor cells in malignant melanoma patients Clin Cancer Res 2004 10 4134 4139 10.1158/1078-0432.CCR-03-0408 15217950

[B16] Eide N Faye RS Hoifodt HK Overgaard R Jebsen P Kvalheim G Fodstad O Immunomagnetic detection of micrometastatic cells in bone marrow in uveal melanoma patients Acta Ophthalmol 2009 87 830 836 10.1111/j.1755-3768.2008.01378.x 19055657

[B17] Tveito S Maelandsmo GM Hoifodt HK Rasmussen H Fodstad O Specific isolation of disseminated cancer cells: a new method permitting sensitive detection of target molecules of diagnostic and therapeutic value Clin Exp Metastasis 2007 24 317 327 10.1007/s10585-006-9052-8 17530423

[B18] Hardingham JE Kotasek D Farmer B Butler RN Mi JX Sage RE Dobrovic A Immunobead-PCR: a technique for the detection of circulating tumor cells using immunomagnetic beads and the polymerase chain reaction Cancer Res 1993 53 3455 3458 8101760

[B19] Trzpis M McLaughlin PM de Leij LM Harmsen MC Epithelial cell adhesion molecule: more than a carcinoma marker and adhesion molecule Am J Pathol 2007 171 386 395 10.2353/ajpath.2007.070152 17600130 PMC1934518

[B20] van der Gun BT Melchers LJ Ruiters MH de Leij LF McLaughlin PM Rots MG EpCAM in carcinogenesis: the good, the bad or the ugly Carcinogenesis 2010 31 1913 1921 10.1093/carcin/bgq187 20837599

[B21] Cimino A Halushka M Illei P Wu X Sukumar S Argani P Epithelial cell adhesion molecule (EpCAM) is overexpressed in breast cancer metastases Breast Cancer Res Treat 2010 123 701 708 10.1007/s10549-009-0671-z 20012351 PMC3042397

[B22] Sieuwerts AM Kraan J Bolt J van der Spoel P Elstrodt F Schutte M Martens JW Gratama JW Sleijfer S Foekens JA Anti-epithelial cell adhesion molecule antibodies and the detection of circulating normal-like breast tumor cells J Natl Cancer Inst 2009 101 61 66 19116383 10.1093/jnci/djn419 PMC2639293

[B23] Vlad AM Kettel JC Alajez NM Carlos CA Finn OJ MUC1 immunobiology: from discovery to clinical applications Adv Immunol 2004 82 249 293 14975259 10.1016/S0065-2776(04)82006-6

[B24] Eaton MC Hardingham JE Kotasek D Dobrovic A Immunobead RT-PCR: a sensitive method for detection of circulating tumor cells Biotechniques 1997 22 100 105 8994656 10.2144/97221st01

[B25] Norwegian breast cancer group http://nbcg.no/

[B26] Delahaye M van der Ham F van der Kwast TH Complementary value of five carcinoma markers for the diagnosis of malignant mesothelioma, adenocarcinoma metastasis, and reactive mesothelium in serous effusions Diagn Cytopathol 1997 17 115 120 10.1002/(SICI)1097-0339(199708)17:2<115::AID-DC6>3.0.CO;2-F 9258618

[B27] Flatmark K Bjornland K Johannessen HO Hegstad E Rosales R Harklau L Solhaug JH Faye RS Soreide O Fodstad O Immunomagnetic detection of micrometastatic cells in bone marrow of colorectal cancer patients Clin Cancer Res 2002 8 444 449 11839662

[B28] Universal ProbeLibrary Assay Design Center https://www.roche-applied-science.com/sis/rtpcr/upl/index.jsp?id=uplct_030000

[B29] Zehentner BK Carter D Mammaglobin: a candidate diagnostic marker for breast cancer Clin Biochem 2004 37 249 257 10.1016/j.clinbiochem.2003.11.005 15003725

[B30] Wang Z Hao Y Lowe AW The adenocarcinoma-associated antigen, AGR2, promotes tumor growth, cell migration, and cellular transformation Cancer Res 2008 68 492 497 10.1158/0008-5472.CAN-07-2930 18199544

[B31] Amiry N Kong X Muniraj N Kannan N Grandison PM Lin J Yang Y Vouyovitch CM Borges S Perry JK Mertani HC Zhu T Liu D Lobie PE Trefoil factor-1 (TFF1) enhances oncogenicity of mammary carcinoma cells Endocrinology 2009 150 4473 4483 10.1210/en.2009-0066 19589871

[B32] Miksicek RJ Myal Y Watson PH Walker C Murphy LC Leygue E Identification of a novel breast- and salivary gland-specific, mucin-like gene strongly expressed in normal and tumor human mammary epithelium Cancer Res 2002 62 2736 2740 12019145

[B33] Thiery JP Epithelial-mesenchymal transitions in development and pathologies Curr Opin Cell Biol 2003 15 740 746 10.1016/j.ceb.2003.10.006 14644200

[B34] Thiery JP Acloque H Huang RY Nieto MA Epithelial-mesenchymal transitions in development and disease Cell 2009 139 871 890 10.1016/j.cell.2009.11.007 19945376

[B35] Kalluri R Weinberg RA The basics of epithelial-mesenchymal transition J Clin Invest 2009 119 1420 1428 10.1172/JCI39104 19487818 PMC2689101

[B36] Putz E Witter K Offner S Stosiek P Zippelius A Johnson J Zahn R Riethmuller G Pantel K Phenotypic characteristics of cell lines derived from disseminated cancer cells in bone marrow of patients with solid epithelial tumors: establishment of working models for human micrometastases Cancer Res 1999 59 241 248 9892213

[B37] Chlenski A Cohn SL Modulation of matrix remodeling by SPARC in neoplastic progression Semin Cell Dev Biol 2010 21 55 65 10.1016/j.semcdb.2009.11.018 19958839

[B38] Mani SA Guo W Liao MJ Eaton EN Ayyanan A Zhou AY Brooks M Reinhard F Zhang CC Shipitsin M Campbell LL Polyak K Brisken C Yang J Weinberg RA The epithelial-mesenchymal transition generates cells with properties of stem cells Cell 2008 133 704 715 10.1016/j.cell.2008.03.027 18485877 PMC2728032

[B39] Tamaki Y Akiyama F Iwase T Kaneko T Tsuda H Sato K Ueda S Mano M Masuda N Takeda M Tsujimoto M Yoshidome K Inaji H Nakajima H Komoike Y Kataoka TR Nakamura S Suzuki K Tsugawa K Wakasa K Okino T Kato Y Noguchi S Matsuura N Molecular detection of lymph node metastases in breast cancer patients: results of a multicenter trial using the one-step nucleic acid amplification assay Clin Cancer Res 2009 15 2879 2884 10.1158/1078-0432.CCR-08-1881 19351770

[B40] Martin Martinez MD Veys I Majjaj S Lespagnard L Schobbens JC Rouas G Filippov V Noterman D Hertens D Feoli F Bourgeois P Durbecq V Larsimont D Nogaret JM Clinical validation of a molecular assay for intra-operative detection of metastases in breast sentinel lymph nodes Eur J Surg Oncol 2009 35 387 392 10.1016/j.ejso.2008.05.008 18639429

[B41] Viale G Dell'Orto P Biasi MO Stufano V De Brito Lima LN Paganelli G Maisonneuve P Vargo JM Green G Cao W Swijter A Mazzarol G Comparative evaluation of an extensive histopathologic examination and a real-time reverse-transcription-polymerase chain reaction assay for mammaglobin and cytokeratin 19 on axillary sentinel lymph nodes of breast carcinoma patients Ann Surg 2008 247 136 142 10.1097/SLA.0b013e318157d22b 18156933

[B42] Effenberger KE Borgen E Eulenburg CZ Bartkowiak K Grosser A Synnestvedt M Kaaresen R Brandt B Nesland JM Pantel K Naume B Detection and clinical relevance of early disseminated breast cancer cells depend on their cytokeratin expression pattern Breast Cancer Res Treat 2011 125 729 738 10.1007/s10549-010-0911-2 20449649

[B43] Oncolex database http://oncolex.no

[B44] Weckermann D Polzer B Ragg T Blana A Schlimok G Arnholdt H Bertz S Harzmann R Klein CA Perioperative activation of disseminated tumor cells in bone marrow of patients with prostate cancer J Clin Oncol 2009 27 1549 1556 10.1200/JCO.2008.17.0563 19237635

[B45] Husemann Y Geigl JB Schubert F Musiani P Meyer M Burghart E Forni G Eils R Fehm T Riethmuller G Klein CA Systemic spread is an early step in breast cancer Cancer Cell 2008 13 58 68 10.1016/j.ccr.2007.12.003 18167340

[B46] Pachmann K Camara O Kavallaris A Krauspe S Malarski N Gajda M Kroll T Jorke C Hammer U Altendorf-Hofmann A Rabenstein C Pachmann U Runnebaum I Hoffken K Monitoring the response of circulating epithelial tumor cells to adjuvant chemotherapy in breast cancer allows detection of patients at risk of early relapse J Clin Oncol 2008 26 1208 1215 10.1200/JCO.2007.13.6523 18323545

[B47] Watson MA Ylagan LR Trinkaus KM Gillanders WE Naughton MJ Weilbaecher KN Fleming TP Aft RL Isolation and molecular profiling of bone marrow micrometastases identifies TWIST1 as a marker of early tumor relapse in breast cancer patients Clin Cancer Res 2007 13 5001 5009 10.1158/1078-0432.CCR-07-0024 17785550 PMC2680916

[B48] Woelfle U Breit E Zafrakas K Otte M Schubert F Muller V Izbicki JR Loning T Pantel K Bi-specific immunomagnetic enrichment of micrometastatic tumour cell clusters from bone marrow of cancer patients J Immunol Methods 2005 300 136 145 10.1016/j.jim.2005.03.006 15907331

[B49] Riethdorf S Fritsche H Muller V Rau T Schindlbeck C Rack B Janni W Coith C Beck K Janicke F Jackson S Gornet T Cristofanilli M Pantel K Detection of circulating tumor cells in peripheral blood of patients with metastatic breast cancer: a validation study of the CellSearch system Clin Cancer Res 2007 13 920 928 10.1158/1078-0432.CCR-06-1695 17289886

[B50] Allard WJ Matera J Miller MC Repollet M Connelly MC Rao C Tibbe AG Uhr JW Terstappen LW Tumor cells circulate in the peripheral blood of all major carcinomas but not in healthy subjects or patients with nonmalignant diseases Clin Cancer Res 2004 10 6897 6904 10.1158/1078-0432.CCR-04-0378 15501967

[B51] Cristofanilli M The biological information obtainable from circulating tumor cells Breast 2009 18 Suppl 3 S38 40 19914540 10.1016/S0960-9776(09)70270-X

[B52] Rao CG Chianese D Doyle GV Miller MC Russell T Sanders RA Jr Terstappen LW Expression of epithelial cell adhesion molecule in carcinoma cells present in blood and primary and metastatic tumors Int J Oncol 2005 27 49 57 15942643

[B53] Pantel K Alix-Panabieres C Riethdorf S Cancer micrometastases Nat Rev Clin Oncol 2009 6 339 351 10.1038/nrclinonc.2009.44 19399023

[B54] Aktas B Tewes M Fehm T Hauch S Kimmig R Kasimir-Bauer S Stem cell and epithelial-mesenchymal transition markers are frequently overexpressed in circulating tumor cells of metastatic breast cancer patients Breast Cancer Res 2009 11 R46 10.1186/bcr2333 19589136 PMC2750105

[B55] Raimondi C Gradilone A Naso G Vincenzi B Petracca A Nicolazzo C Palazzo A Saltarelli R Spremberg F Cortesi E Gazzaniga P Epithelial-mesenchymal transition and stemness features in circulating tumor cells from breast cancer patients Breast Cancer Res Treat 2011 in press 21298334 10.1007/s10549-011-1373-x

[B56] Sorlie T Perou CM Tibshirani R Aas T Geisler S Johnsen H Hastie T Eisen MB van de Rijn M Jeffrey SS Thorsen T Quist H Matese JC Brown PO Botstein D Eystein Lonning P Borresen-Dale AL Gene expression patterns of breast carcinomas distinguish tumor subclasses with clinical implications Proc Natl Acad Sci USA 2001 98 10869 10874 10.1073/pnas.191367098 11553815 PMC58566

[B57] Perou CM Sorlie T Eisen MB van de Rijn M Jeffrey SS Rees CA Pollack JR Ross DT Johnsen H Akslen LA Fluge O Pergamenschikov A Williams C Zhu SX Lonning PE Borresen-Dale AL Brown PO Botstein D Molecular portraits of human breast tumours Nature 2000 406 747 752 10.1038/35021093 10963602

[B58] Park SY Lee HE Li H Shipitsin M Gelman R Polyak K Heterogeneity for stem cell-related markers according to tumor subtype and histologic stage in breast cancer Clin Cancer Res 16 876 887 10.1158/1078-0432.CCR-09-1532 PMC2818503 20103682

[B59] Tomaskovic-Crook E Thompson EW Thiery JP Epithelial to mesenchymal transition and breast cancer Breast Cancer Res 2009 11 213 10.1186/bcr2416 19909494 PMC2815537

