# Supplementary material for: Analysis of EpCAM positive cells isolated from sentinel lymph nodes of breast cancer patients identifies subpopulations of cells with distinct transcription profiles
Source: Breast Cancer Res. 2011 Aug 4;13(4):R75. doi: 10.1186/bcr2922 (PMC3236339; doi:10.1186/bcr2922)
Supplement: Additional file 1 — Primers and probes used for RT-PCR. Sequence of primers designed using ProbeFinder from Roche Applied Science and the corresponding probes from the Universal ProbeLibrary [28] [file bcr2922-S1.DOC]

Supplementary file 1. Primers and probes used for RT-PCR

| **Target** | **Primers** | **Universal probe** | **NCBI number** | **Amplicon length** |
| --- | --- | --- | --- | --- |
| **YARS** | F: 5’GGATTAACAGGCAGCAAAATG | # 35 | NM_003680.2 | 67 nt |
|  | R: 5’CCTTCCGATCAAGGAGATCA |  |  |  |
| **hMAM** | F: 5’CTCCCAGCACTGCTACGC | # 71 | NM_002411.2 | 72 nt |
|  | R: 5’TGTGGATTGATTGTCTTGGAAA |  |  |  |
| **AGR2** | F: 5’GGTGGGTGAGGAAATCCAG | # 47 | NM_006408.2 | 68 nt |
|  | R: 5’GGCCACAAGGAGCAAGAAT |  |  |  |
| **SBEM** | F: 5’GCCCAGAATCCGACAACA | # 18 | NM_058173.2 | 90 nt |
|  | R: 5’AGCAGTGGTTTCAGCATCAG |  |  |  |
| **TFF1** | F: 5’CCCTCCCAGTGTGCAAATA | # 66 | NM_003225.2 | 131 nt |
|  | R: 5’GATCCCTGCAGAAGTGTCTAAAA |  |  |  |
| **EpCAM** | F: 5’CTCCACGTGCTGGTGTGT | # 3 | NM_002354.1 | 111 nt |
|  | R: 5’TGTTTTAGTTCAATGATGATCCAGTA |  |  |  |
| **Wnt5B** | F: 5’GGAGCGAGAGAAGAACTTTGC | # 82 | NM_032642.2, NM_030775.2 | 107 nt |
|  | R: 5’ CGTCTGCCATCTTATACACAGC |  |  |  |
| **SPARC** | F: 5’GAGGTGGTGGCGGAAAAT | # 42 | NM_003118.2 | 87 nt |
|  | R: 5’GCACATGGGGGTGTTGTT |  |  |  |
| **EGFR** | F: 5’ CATGTCGATGGACTTCCAGA | # 44 | NM_201282.1 | 61 nt |
|  | R: 5’GGGACAGCTTGGATCACACT |  |  |  |
| **Col1A2** | F: 5’CTGGAGAGGCTGGTACTGCT | # 85 | NM_000089.3 | 86 nt |
|  | R: 5’GCCAGGGAGACCCAGAATA |  |  |  |
| **MMP2** | F: 5’ATAACCTGGATGCCGTCGT | # 70 | NM_001127891.1, NM_004530.4 | 63 nt |
|  | R: 5’AGGCACCCTTGAAGAAGTAGC |  |  |  |
| **ERBB2** | F: 5’CAACTGCACCCACTCCTGT | # 85 | NM_001005862.1 | 87 nt |
|  | R: 5’GCAGAGATGATGGACGTCAG |  |  |  |

All primers are designed using ProbeFinder version 2.43 from Roche Applied Science [28].
